# What happened in the ‘Move for Well-being in School’: a process evaluation of a cluster randomized physical activity intervention using the RE-AIM framework

**DOI:** 10.1186/s12966-017-0614-8

**Published:** 2017-11-16

**Authors:** Søren Smedegaard, Ruben Brondeel, Lars Breum Christiansen, Thomas Skovgaard

**Affiliations:** 1 FIIBL, Research and Innovation Centre for Human Movement and Learning, Odense M, Denmark; 2 SDU, University of Southern Denmark, Campusvej 55, 5230 Odense M, Denmark; 3 IOB, Department of Sports Science and Clinical Biomechanics, Campusvej 55, 5230 Odense M, Denmark; 4 UCL, University College Lillebaelt, UCL Campus Odense, Niels Bohrs Allé 1, 5230 Odense M, Denmark; 5 Centre de recherche du CHUM, 900 St Denis St, Montréal, QC H2X 0A9 Canada

**Keywords:** Physical activity, Well-being, School, Process evaluation, Cluster RCT, Implementation, RE-AIM

## Abstract

**Background:**

The aim of this study was to address the gap in the translation of research into practice through an extensive process evaluation of the *Move for Well-being in School* programme using the RE-AIM framework. The purpose was to gain insight into the extent by which the intervention was adopted and implemented as intended and to understand how educators observed its effectiveness and maintenance.

**Methods:**

Public schools located in seven municipalities in Denmark were invited to enroll their 4th to 6th grade classes in the project. Of these, 24 school decided to participate in the project in the school-year 2015–16 and were randomly (cluster) allocated to either intervention or control group. A process survey was completed online by school personnel at the start, at midterm, and at the end of the school year. Additionally, informal interviews and observations were conducted throughout the year.

**Results:**

At the 12 intervention schools, a total of 148 educators were involved in the implementation of the programme over the school-year. More than nine out of ten educators integrated brain breaks in their lessons and practically all the physical education teachers used the physical education lesson plans. The educators delivered on average 4.5 brain breaks per week and up to 90% of the physical education teachers used the project lesson plans for at least half of their classes. Half of the educators initiated new recess activities.

A total of 78%, 85% and 90% of the educators believed that the implemented recess, brain break and physical education components ‘to a high degree’ or ‘to some degree’ promoted the pupils’ well-being, respectively.

**Conclusions:**

This study shows that it is possible to design a school-based PA intervention that educators largely adopt and implement. Implementation of the PA elements was stable throughout the school year and data demonstrate that educators believed in the ability of the intervention to promote well-being among the pupils. Finally, the study show that a structured intervention consisting of competence development, set goals for new practices combined with specific materials, and ongoing support, effectively reached a vast majority of all teachers in the enrolled schools with a substantial impact.

**Trial registration:**

Date of registration: retrospectively registered on 24 April 2015 at Current Controlled Trials (DOI 0.1186/ISRCTN12496336 – named: “The role of physical activity in improving the well-being of children and youth”).

**Electronic supplementary material:**

The online version of this article (10.1186/s12966-017-0614-8) contains supplementary material, which is available to authorized users.

## Background

School-based approaches promoting physical activity (PA) are recommended because in most countries the majority of children and adolescents spend many hours in school every day [1–3]. A school setting also makes it possible to reach children and young people who are fairly inactive during their leisure time. Furthermore, in many countries health and well-being are an integrated part of the state school curriculum, which means that there are qualified educators in the form of teachers, pedagogues and other adults teaching at the school, alongside existing cultures and infrastructures for teaching and learning about activities that relate to health, well-being and PA [1, 4]. Well-being can be defined in different ways, but in the Danish school system, well-being is most often understood in relation to social and academic factors, which are focus areas in the annual ‘school well-being survey’. The understanding of well-being used in this project is based on the self-determination theory, where self-realization and vitality are central aspects [5]. Well-being is enhanced if the three innate psychological needs: autonomy, competence and social relatedness is satisfied [6]. Engaging in physical activity can be more or less conducive for fulfilling the three psychological needs, which is dependent on the context and the social environment [7]. Recently, Lubans and colleagues offered a conceptual model describing three possible mechanisms between PA and various mental health outcomes. The model suggests three hypotheses: a neurobiological; a psychosocial; and a behavioral hypothesis which could explain the rationale and possible impact of PA on well-being [8]. In recent years, a number of school-based interventions have been conducted with a focus on PA and well-being with small to moderate positive effects [9–15]. Many of these studies were small-scale studies; conducted over a relatively short period of time (4–20 weeks intervention); lacking a control group; or used a cross-sectional design [3, 16, 17].

In order to construct evidence applicable to real-world settings, it is crucial to evaluate the feasibility of the intervention programme, and this is no easy task [18]. Several studies have addressed the implementation of physical activity programmes in schools, identifying several determinants of a successful implementation [19, 20]. In a systematic review, Naylor et al. described the most common categories used in previous studies, such as 1) provider characteristics, 2) characteristics of the innovation, 3) the delivery system and 4) the support system, all of which is critical to consider when designing school-based PA interventions [19]. Many programmes however fail to evaluate the process of the interventions, which could be an explanation for their general low effectiveness and, more importantly, could hinder the flow of information to future interventions that might improve implementation mechanisms.

The RE-AIM framework (Reach, Effectiveness, Adoption, Implementation, and Maintenance) has been developed to guide the evaluation of issues relating to the external validity that is, ´finding out which populations it works for and how best to make it work in those populations´ [21]. The framework has been used to assess school-based PA interventions [22–24] and to guide process evaluations in cluster randomized controlled trials [25]. The RE-AIM framework encourages planners, evaluators, and policy-makers to pay more attention to essential programme elements, including external validity. In this way, the sustainable adoption and implementation of effective, generalizable, evidence-based interventions can be improved [26].

The *Move for Well-being in School* intervention (MWS) aimed to improve psychosocial well-being among school-aged children and youths from 4th to 6th grade (10–13 years) through the development, implementation, and evaluation of a multicomponent, school-based, physical activity intervention. The objective of this paper is to evaluate the implementation of MWS from the implementer’s point of view (the educator), using the RE-AIM framework.

## Methods

### The Move for Well-being in School programme

The programme was designed, piloted and implemented in accordance with the study protocol published previously [27]. In brief, the programme consisted of a four-phased intervention – design, pilot, randomized controlled trial (RCT), and evaluation – guided by *The Medical Research Council* [28] framework for the development of complex interventions. A CONSORT checklist for the intervention has been produced and is added as an Additional file 1.

In the design phase, the preliminary development processes entailed conducting a scoping review, interviews with members of the target group and the execution of four workshops including a broad selection of key stakeholders. Informed by the design phase, an initial intervention programme was assembled. Also, the main theoretical driver of the programme development originated from the area of motivation, as construed by Edward Deci and Richard Ryan’s self-determination theory [6]. The pilot phase encompassed the assessment of the initial intervention programme in four schools over a four-month period, to evaluate the feasibility of the implementation in a real-world setting.

**Fig. 1:**
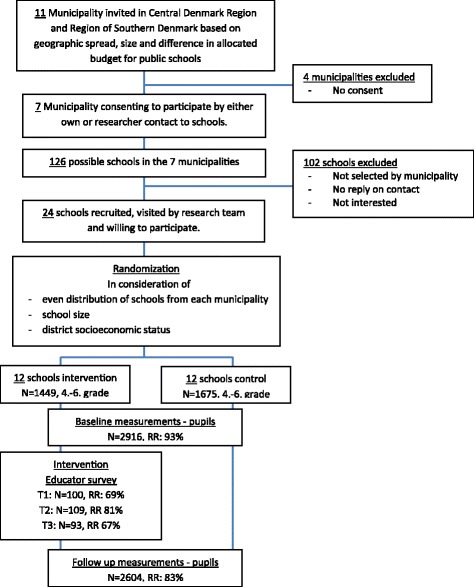
Flowchart of recruitment, randomization, and measures. RR: Response Rate

The RCT included the implementation of the final intervention programme. Based on initial screening municipalities were selected from the following criteria a) geographic and demographic variation, b) variation in schools size and c) variation without extremes in municipal budgets for public schools [29]. A total of 11 of 98 Danish municipalities were contacted and seven agreed to participate either by contacting schools themselves or by allowing the research team to contact schools in the municipality [27]. The research team held individual meetings with all interested schools and a total of 24 schools were enrolled. A stratified randomization was conducted with three strata and with the constraint of an even distribution of schools from each municipality in the intervention group and the control group respectively. The three strata were defined by school typology, based on school size and district socioeconomic status [27], see Fig. 1.

**Fig. 2:**
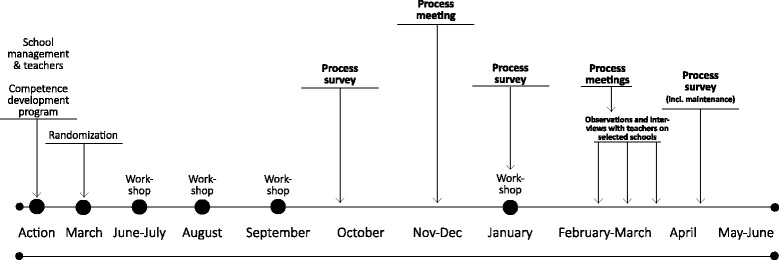
Timeline

The intervention consisted of, (i) a competence development programme for educators consisting of four workshops with both practical and theoretical content (Fig. 2); (ii) inspiration material on the MWS website [30]; and (iii) the coordination between the schools and the research team, primarily via local coordination groups consisting of representatives from 4th, 5th, and 6th grades, often physical education teachers, and a member of the school management [27]. Two process meetings, by the research team, were conducted at each school (Fig. 2). The PA program included physical education, recess, theme days and brain breaks. In short, the educators should conduct two daily brain breaks lasting five minutes per class; facilitate new activities during recess three times per week lasting 30 min; complete three theme days focusing on well-being and PA; and finally, half of physical education classes should be taught according to the special MWS lesson plans designed for the intervention [27].

At the 12 intervention schools, a total of 148 educators were involved in the implementation of the MWS program over the school year of which 48 were PE teachers. The average number of involved educators per school was 12 (range 5–17), and three of these were physical education teachers (range 1–6).

The programme evaluation phase contains a joint evaluation of the entire project period, guided by the RE-AIM framework [26].

### Data collection

Data collection featured administrative data, online surveys, informal interviews, and observations. Administrative data on municipalities and schools were obtained from the Ministry of Economic Affairs and the Interior and from the municipalities. Educators were asked to complete an online questionnaire three times during the intervention: two months (T1), five months (T2) and nine months (T3) after commencement (Fig. 2). At T1, 141 educators answered of whom 42 were PE teachers, at T2, 135 educators answered of whom 48 were PE teachers and at T3, 139 educators answered of whom 39 were PE teachers.

Educators were included in analyses if they responded on at least two of the time points. The questionnaire encompassed items related to the educators, the intervention, the delivery of the intervention, the support system and open ended question related to experienced barriers in the intervention delivery. The three rounds of self-administered online questionnaires were used to collect data on the subsequent RE-AIM components: effectiveness, adoption, implementation, and maintenance [27]. Structured observation of PE lessons and qualitative interviews with PE teacher from four schools were conducted as part a master thesis which were part of the project [31].

Finally, during the process meetings (Fig. 2), the research team retrieved information on implementation progress, barriers, and facilitators. Informal interviews with coordination groups focused on statements related to the role of the CG [27]. Data from observation and interviews with PE teachers and open ended answers from the questionnaire is included in the discussion section to add insights and perspectives.

### RE-AIM elements (Table 1)

#### Reach

Since the 24 participating schools can be described as a convenience sample, the ‘reach’ of the intervention is understood as the representativeness of the schools compared to the national average. As part of ‘reach’ evaluation, we looked at characteristics of the schools to ensure representativeness in relation to the number of educators and PE teachers.

**Table 1:** Evaluation dimensions and definitions of RE-AIM

Evaluation Dimensions definitions of RE-AIM	
*Dimension*	*Definitions*
Reach	Refers to the proportion and representativeness of eligible schools willing to participate in the study.
Effectiveness	Is operationalized at the educators’ perceptions of the degree to which the three physical activity intervention components influenced child well-being.
Adoption	Refers to the educator’s decision to adopt the PA intervention or not. Reported as the proportion of educators who delivered any part of the PA intervention.
Implementation	Refers to the educators’ fidelity to the various elements of the intervention’s protocol, including the implementation quality and the consistency of delivery as intended.
Maintenance	Refers to the extent to which intervention implementation was maintained throughout the school year – short-term maintenance. Additionally, indicators of sustainability were identifying at the educator level.

**Table 2:** Comparison of intervention and control schools to the national average

Schools average	Boys (%)	School size (school/4.-6.th grade)	Family social class High/medium/low	Avg. gross revenue^c^ (× 1000 kr^d^.)	Expenses per pupil^e^(× 1000 kr.)
Intervention	50.7	435 / 138	43.0 / 46.3 / 10.7	650	63.7
Control	51.5	490 / 154	39.2 / 48.0 / 12.8	617	64.0
DK average^a^	51.1	421 / -	44.6/44.6/10.8^b^	666	69.0

^a^DK average is based on statistics from all Danish schools from Ministry of Economic Affairs and the Interior

^b^Based on 4534 pupils from 5. to 9.th grade in 48 schools distributed in all regions of Denmark, 11% not classified, 5% economic inactive [48]

^c^Parents’ average yearly income. If parents are divorced, data are generated from the cohabiting adults and not the biological parents

^d^Danish kroner (1 kr = 0.14 USD/0.13 Euro)

^e^Expenses are net expenses for the public school

Schools’ representativeness was assessed using data on gross revenue from the schools and expenses per pupil, retrieved from the Ministry of Social Affairs and the Interior, and information on municipalities’ budget and budget prerequisites. Data on family social class, collected and coded from the pupil’s survey [27], is compared to data from the Danish contribution to the international study on family social class, *Health Behaviour in School-aged Children* [32, 33].

#### Effectiveness

Most commonly effectiveness is evaluated as the impact of an intervention on primary outcome measured at end-user level, but due to the implemetation focus in this study, we used the educators’ perceptions of effectiveness of the intervention. Educators were asked to what degree they believed in an effect of the intervention components on pupils’ well-being; and whether they had noticed an improvement in pupil well-being due to the project as a whole. In this way, we capture the educator’s perception, which is highly relevant in process evaluations at this level and has an influence on the degree of implementation and maintenance [34]. The effect evaluation of potential outcomes on end-user level is addressed elsewhere [35].

#### Adoption

Despite the consent from school management to participate, there was no guarantee that the educators would adopt the program. Educators could be reluctant to change and continue their normal practice without consequences. Adoption of the program was operationalised at the school level as the proportion of educators who reported conducting any elements of the MWS at the three time-points.

#### Implementation

Implementation refers to the intervention agents’ fidelity to the various elements of the intervention’s protocol, including the consistency of delivery as intended and the implementation quality.

Fidelity was measured for the three main PA components. Each class should receive two brain breaks each day, and educators were asked how many brain breaks they did in average for each class. Data were summarised at class level. Regarding physical education, six of the eight tailored MWS physical education courses should be part of the curriculum. This equals approximately half of the available lessons. These data were summarised at year level. For recess, the educators should actively facilitate activities during recess three days per week. Teachers were asked for how many days they initiated activities or helped pupils initiate activities. Data were aggregated on school level because educators are responsible for all three-year levels during recess. Additionally, as an indicator of implementation quality, the educators were asked to rate their level of preparedness in working with physical education, recess and brain breaks in the project [27].

##### Maintenance

The schools were followed for an entire school year, and the final survey (T3) was conducted at the end of the school year – four months after the last competence development programme workshop (Fig. 2). This continuation of programme delivery until the last process survey is defined as short-term maintenance. Based on literature review additional indicators of intervention sustainability were operationalized and asked at T3 [19]. The educators were asked to rate questions regarding school priorities; the adaptability of the program to their everyday work day; and whether the approach in the project was consistent with their self-image as educators. Finally, two questions on whether they would recommend MWS to other schools and overall effect at school level were asked.

## Results

The process surveys were completed by 100 of 141 educators at Time 1(T1) (69%, range 47–100%), by 109 of 135 possible educators at Time 2 (T2) (81%, range 60–100%) and finally, 93 of 139 possible educators at Time 3 (T3) (67%, range 50–88%), averaging as a 72% response rate. Only educators that completed at least two process surveys were included in the analyses.

### Reach

Table 2 compares school characteristics from the intervention and control groups with national averages. The families from the enrolled schools have a little lower gross revenue compared to the Danish average, which is more profound for the families at the control schools. The expenses per pupil are lower at the enrolled schools, and the control schools are a little larger compared to intervention schools and the national average.

### Effectiveness

**Fig. 3:**
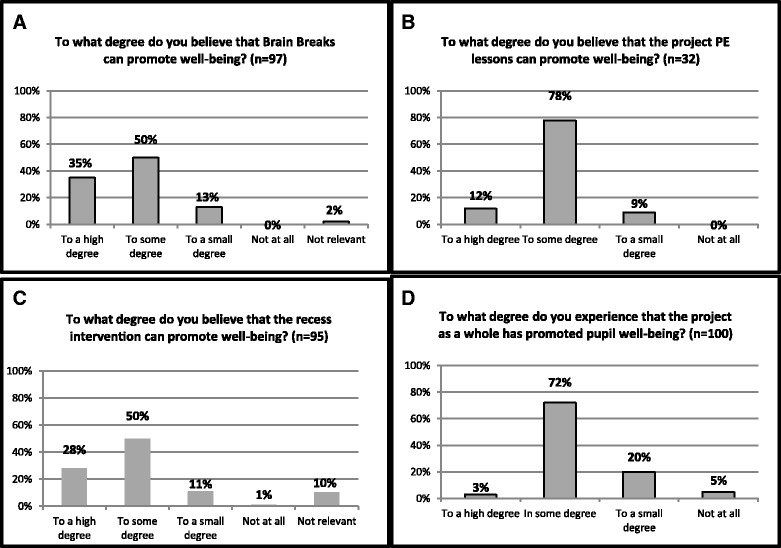
**a**-**d** – Believe that activities and project improved well-being

At T3, more than eight out of ten educators believe that brain breaks improved pupils’ well-being ‘to a high degree’ or ‘to some degree’ (Fig. 3a). As for the physical education teachers, 12% reported that the physical education lessons designed for this project improved the pupils’ well-being ‘to a high degree’, while close to 80% reported this to be the case ‘to some degree’ (Fig. 3b). Recess initiatives improved pupils’ well-being ‘to a high degree’ according to 28% of the educators at T3, and ‘to some degree’ according to 50% of the educators (Fig. 3c).

Finally, when asked about the impact of the physical activity interventions as such, 75% of the educators answer that this improved well-being among their pupils ‘to a high degree’ or ‘to some degree’, (Fig. 3d).

### Adoption

More than nine out of ten educators integrated brain breaks in their lessons, and all physical education teachers used the lesson plans at least once (Table 3). Around half of the educators initiated activities during recess.

**Table 3:** Proportion of educators that did part of the PA interventions

	Brain break proportion who did any within a typical week (n: T1 = 98, T2 = 111, T3 = 97)		Physical education proportion who did at least one lesson as MWS-lessons (n: T1 = 38, T2 = 45, T3 = 32)		Recess proportion who initiate activities at least once within a typical week (n: T1 = 97, T2 = 110, T3 = 95)	
	Average (%)	School range (%)	Average (%)	School range (%)	Average (%)	School range (%)
T1	94	80–100	100	–	57	17–86
T2	95	83–100	98	75–100	48	17–100
T3	91	75–100	100	–	49	36–100
Avg.	93	75–100	99	75–100	51	16–100

### Implementation

The set goal for the intervention was: two brain breaks per day; approximately half of the physical activity lessons organized according to the MWS programme; and initiated/facilitated recess activities three times a week lasting at least 30 min each [27].

On average, the educators delivered 4.1, 4.5 and 4.8 brain breaks per week at T1, T2, and T3 respectively. There were large differences between schools. Teachers at the school with the lowest implementation conducted approximately three brain breaks, while teachers at the schools with the highest implementation conducted twice as many (Table 4). The average number of brain breaks per class per week is estimated multiplying the average 4.5 brain breaks/week with the average 138 educators, divided by the 72 enrolled classes. This gives an overall average of 8.6 brain breaks per class per week if the non-responding educators did as many brain breaks as the responders.

**Table 4:** Numbers, percentages and days of brain breaks, physical education and recess delivered

	Brain break, number per week (n: T1 = 98, T2 = 111, T3 = 97)		Physical Education, % who followed the MWS PE-program for at least half of the PE lessons (n: T1 = 38, T2 = 45, T3 = 32)		Recess, number of days of educator initiated activities (n: T1 = 97, T2 = 110, T3 = 95)	
Variable	Average	School range	%	School range	Average	School range
T1	4.1	2.8–6.1	80	33–100	1.6	1–2.5
T2	4.5	3.2–5.9	90	50–100	1.6	1–5
T3	4.8	3.1–6.3	90	50–100	1.6	1–2.5
Avg.	4.5	2.8–6.3	83.3	33–100	1.6	1–5

Between 80% and 90% of the physical education teachers used the project lesson plans for at least half of their physical education classes with variation between schools. Finally, there were no differences between the three time-points for recess activities initiated.

As for a more general implementation quality indicator, we asked: “*To what degree do you feel prepared to work with physical education/brain breaks/recess in the project?”*

Table 5 show that educators to a large extent perceived themselves as prepared for working with the project’s physical activity elements. Approximately 95% of the educators felt that they ‘to a high degree’ or ‘to some degree’ were prepared for the brain breaks part. The educators felt more prepared for the new physical education practice at the end of the school year. At T1 around 80% indicated being prepared ‘to high degree’ or ‘to some degree’. This number increased to more than 90% at T2, and 100% at T3. Recess was the area with the lowest level of self-perceived preparedness. The percentage of educators feeling prepared ‘to a small degree’ increased from 6% to more than 23% from the first to the last process survey. At the same time, there was nearly a 20% decrease in educators answering that they felt prepared ‘to some degree’.

**Table 5:** Distribution of answers to the question on preparedness

	Preparedness for brain breaks *(n: T1 = 98, T2 = 111, T3 = 97)*				Preparedness for physical education *(n: T1 = 38, T2 = 45, T3 = 32)*				Preparedness for Recess activities *(n: T1 = 97, T2 = 110, T3 = 95)*				
Response categories^a^	*1*	*2*	*3*	*4*	*1*	*2*	*3*	*4*	*1*	*2*	*3*	*4*	*5*
T1 (%)	45.1	50.4	1.8	2.7	22.0	56.1	19.5	2.4	11.8	63.6	6.4	2.7	15.5
T2 (%)	50.0	44.4	4.0	1.6	33.3	58.3	8.3	0	11.4	51.2	17.9	0	18.7
T3 (%)	40.2	55.9	3.9	0	28.1	71.9	0	0	10.8	44.1	23.5	2.0	19.6

Question: To what degree do you feel prepared to work with PE/brain breaks/recess?

^a^Response categories: 1 = *to a high degree,* 2 = *to some degree,* 3 = t*o a small degree,* 4 = *not at all,* 5: *not relevant (some educators were omitted for recess duty)*

The project intended to increase the feeling of preparedness by having the educators participate in the competence development programme. The workshops had a high participation rate, as the majority of educators attended at least one workshop. Collectively, 85% of the educators, that completed the surveys, participated in at least one workshop. The participation rate per school ranged from 70 to 100% of the educators participating at least once – except for one school that had a 29% participation rate. A total of 95% of the physical education teachers participated at least once in a workshop, while only two schools had physical education teachers who did not participate. Finally, 79% of the educators participated in the recess workshop, which was held at the local schools. Participation rates ranged from 45% to 100% between the participating schools.

### Maintenance

As seen in Tables 3 and 4 the change over time in delivered activities was very small. There was a minor decrease from T1 to T3 in the proportion of educators doing any brain breaks (94% to 91%), but on the contrary, the educators who did brain breaks, did a little more. The question regarding physical education asked about the proportion of physical education devoted to MWS during the whole school year. Thus, it is not possible to detect if they increased or decreased the use of the MWS-lessons. As for recess, a decline between educators initiating recess activities between T1 and T3 (57% to 49%) was observed. However, the compliant educators maintained the number of days at 1.6 days per week. All in all, the overall trend of implementation did not seem to decrease throughout the school year.

In the final survey, the educators were asked about five indicators of programme sustainability. As seen in Tables 6, 85% of the educators reported that the project fits the school’s other priorities ‘to a high degree’ or ‘to some degree’. A total of 93% answered that PA, and movement in general, is prioritized at the school ‘to a high degree’ or ‘to some degree’. Asked whether the initiatives in the project were adaptable to their everyday work, 16% answered ‘to a high degree’ and 70% ‘to some degree’. The clear majority of educators answered that the project was favourable compared to other initiatives, and they found it to be consistent with their own self-image as educators. Finally, approximately nine out of ten educators would recommend MWS to other schools and felt that participation in the project had improved their school (data not shown).

**Table 6:** Distribution of answers to the question: “To what degree do you experience, ..” at T3 (*n* = 93)

	To a high degree (%)	To some degree (%)	To a small degree (%)	Not at all (%)
*..the project fits the school’s other priorities*	23.7	61.3	11.8	3.2
*..PA, and movement in general, are prioritized in the school*	32.3	60.2	7.5	–
*..the initiatives in the project are adaptable to their everyday work*	16.1	69.9	12.9	1.1
*..the project was favorable compared to other initiatives*	26.9	63.4	7.5	2.2
*..consistent with their own self-image as educators*	31.2	57.0	10.7	1.1

## Discussion

The objective of this study was to evaluate the implementation of Move for Well-being in School using the RE-AIM framework. Often, as is the case with MWS, interventions address several outcomes simultaneously and are comprised of a number of components that interact and affect outcomes. Moore et al. [36] remarked that interventions are often delivered in systems that are complex and respond unpredictably. In order to contain this complexity, intervention evaluations have to move beyond a focus on effectiveness alone. Process evaluation is one way to investigate other important dimensions in health interventions.

### Reach

As stated in the results section, schools were selected to ensure comparability to most Danish schools in terms of number of pupils, expenses per pupil, and socioeconomic status of the pupil’s parents. We included 24 schools but had difficulties reaching schools through the municipal authorities. Municipalities were reluctant to put pressure on schools due to a recent extensive school reform and consequently increased workload. Therefore, with permission from the municipalities, the schools were contacted directly. The schools agreeing to participate could be grouped into two basic categories: 1) schools that already had a consolidated focus on school PA and were interested in improving the already high standards; and 2) schools with low experience and capabilities regarding school PA and with obvious challenges meeting the target of 45 min of school PA per day. This variation could contribute to understanding the differences in school management and educators’ motivation for participating in the project and capacities for taking on MWS. According to Scaccia et al. the ability to take on a particular innovation depends on “*a) the motivation to implement an innovation, b) the general capacities of an organization, and c) the innovation-specific capacities needed for a particular innovation”*, also referred to as organisational readiness [37]. The ‘organisational readiness’ could be reflected in the response rates, which showed that some schools only achieved answers from half of the educators, while other schools had a response rate of 100%. Presumably, non-responding educators were less involved in the programme and implemented fewer activities and components. The differences in response rates between schools might, therefore, bias the results.

### Effectiveness

As an indicator of effectiveness, we used the educators’ perception of the pupils’ change in well-being. The definition of well-being and introduction to the key elements of the self-determination theory was presented in the available materials and during the competence development program. Still, it is uncertain whether the educators’ perceptions accurately reflect actual changes in pupils’ well-being. Nonetheless, the overall belief in the positive effects of the intervention is evident and it is essential to the educators’ motivation to implement and maintain the programme.

### Adoption

The adoption rates for both physical education and brain breaks were high, but the adoption rate of the recess activities was much lower, which may reflect the fact that not all educators are appointed for recess duty. Recess is used by many educators as a time for preparation and coordination or having a break and an informal talk with colleagues. Having ‘recess duty’ is tantamount to just ensuring pupils are not getting in trouble or injured. Some teachers also hold the view, that recess is free time for the pupils and should not be influenced in any way by the adults.

The recess activities could, therefore, be experienced as a rather radical change compared to previous practices in the area. In general, the literature holds that the more radical a change is, the more uncertainty it creates and the more difficult the implementation is [38]. Whereas the educators generally found it difficult to intervene during recess due to time constraints, the challenges for brain breaks revolved around spending time away from the key academic subject. Two reasons why adoption in physical education might be less challenging is that physical education teachers have PA competences and education, and secondly that the physical education lessons should be conducted anyway [39]. In that sense the physical education component and the lesson plans could be regarded as a help to an existing task and not as extra work, which was stated by some of the PE teachers involved in the competence development program. This finding is also supported in the proces evaluation by Steeinhuis et al., where an intervention that involved extra work on top of a heavy workload of regular duties were seen as a barrier for implementation [40].

The fact that no school withdrew from the project, together with the high response rates for the process survey, lends confidence in the representativeness of the presented data. The role of the coordination groups at each school consisting of educators and management representatives could partly explain the high participation and adoption rate. Active management participation and involvement have in previously process evaluations been stated as facilitator for engaging in project interventions [41].

### Implementation

The implementation of brain breaks was 4.5 per educator per week with major differences between educators and an overall average of 8.6 brain breaks per class per week for all intervention schools. Barriers for conducting brain breaks relates to difficulties integrating it to the normal practice. Finally, some brain breaks lasted more than 5 min, which might let the educators settle for one per day of longer duration.

Overall, the physical education set goal was met, with at least half of physical education lessons being MWS lessons. This was probably because physical education teachers perceived it as a help to their planning, and because this element is directed at teaching and learning and thus resembles normal school practice and is related to the academic subject [39, 42] . Interventions that align directly with a school’s mission are easier to integrate into the school’s policy and practices and are more likely to be prioritised, implemented with due care and quality, and sustained over time [43].

The observations of and interviews with the physical education teachers supported the findings that the MWS lessons plans were positively received. They also indicated that lack of time for preparation; lack of coordination between teachers; and challenges with some pupils’ acceptance of the new physical education practice were among the biggest barriers to the implementation [31]. The physical education teachers that attended the workshops emphasised these as highly significant for their preparation and for their motivation for the project. The educators stated that the practical learning of brain breaks and physical education, and the opportunity to meet with their colleagues were pivotal [31]. Similar findings are reported by Castelli et al. in their examination of the strength of evidence from studies of professional development effectiveness [44].

Several studies have previously reported barriers and facilitators in implementing school-based PA interventions [19, 41]. Answers from the open ended question in the process survey on “W*here do you experience the biggest challenges in implementing the intervention components?”* confirm the general findings in the process literature [19]. ‘Lack of time’ and ‘time for preparation’ being the most common answers with 26 unique comments out of collectively 33 comments. The remaining comments revolves around change in PE culture with four comments, challenges in recess with four comments and finally one comment on ‘lack of commitment among colleagues’, ‘pupils avoid academic for more brain break time’ and ‘noise during brain breaks’ respectively. These factors affecting implementation in MWS matched the categories by Nayor et al. presented earlier. The two most common facilitators in the project were the competence development program and the opportunity for local tailoring [19, 41, 45, 46].

### Maintenance

Both adoption and implementation are relatively stable between T1 and T3. During the school year the coordination group received bi-weekly information letters by e-mail; received two follow-up visits from the research team; conducted a mid-term theme day; and were invited to attend the fourth workshop half way through the school year. The relatively low level of input from the research team required for ongoing implementation provided an important enticement for the maintenance of the MWS initiatives. Furthermore, the fact that the intervention was conducted over a whole school year and employed teacher-delivered strategies is in the process evaluation literature perceived as facilitators for increasing maintenance [47]. Finally, the fact that nine out of ten educators would recommend the MWS programme to other schools and felt that participation had improved their school, lead us to believe that the intervention can be maintained over time [19, 41].

## Conclusion

This study have shown that it is possible to design a school-based PA intervention that educators largely adopt and implement. Implementation of the PA elements was stable throughout the school year and data demonstrate that educators believed in the ability of the intervention to promote well-being among the pupils. There were, however, large differences between schools in implementation, which can be explained by differences in existing capabilities and motivation. Finally, the MWS show that a structured intervention consisting of competence development, set goals for new practices combined with specific materials, and ongoing support, effectively reached a vast majority of all teachers in the enrolled schools with a substantial impact.

## Additional file

Additional file 1: CONSORT checklist. (DOCX 58 kb)

## Abbreviations

BB: Brain Breaks
MWS: Move for Wellbeing in School
PA: Physical Activity
PE: Physical Education
RCT: Randomized Controlled Trial
RE: Recess
